# Epigenetic Regulation of EMT (Epithelial to Mesenchymal Transition) and Tumor Aggressiveness: A View on Paradoxical Roles of KDM6B and EZH2

**DOI:** 10.3390/epigenomes3010001

**Published:** 2018-12-20

**Authors:** Camille Lachat, Michaël Boyer-Guittaut, Paul Peixoto, Eric Hervouet

**Affiliations:** 1 Interactions Hôte-Greffon-Tumeur/Ingénierie Cellulaire et Génique, INSERM, EFS BFC, UMR1098, Univ. Bourgogne Franche-Comté, F-25000 Besançon, France; 2 DImaCell Platform, Univ. Bourgogne Franche-Comté, F-25000 Besançon, France; 3 EPIGENEXP Platform, Univ. Bourgogne Franche-Comté, F-25000 Besançon, France

**Keywords:** cancer, EMT, metastasis, epigenetics, KDM6B, EZH2, H3K27

## Abstract

EMT (epithelial to mesenchymal transition) is a plastic phenomenon involved in metastasis formation. Its plasticity is conferred in a great part by its epigenetic regulation. It has been reported that the trimethylation of lysine 27 histone H3 (H3K27me3) was a master regulator of EMT through two antagonist enzymes that regulate this mark, the methyltransferase EZH2 (enhancer of zeste homolog 2) and the lysine demethylase KDM6B (lysine femethylase 6B). Here we report that EZH2 and KDM6B are overexpressed in numerous cancers and involved in the aggressive phenotype and EMT in various cell lines by regulating a specific subset of genes. The first paradoxical role of these enzymes is that they are antagonistic, but both involved in cancer aggressiveness and EMT. The second paradoxical role of EZH2 and KDM6B during EMT and cancer aggressiveness is that they are also inactivated or under-expressed in some cancer types and linked to epithelial phenotypes in other cancer cell lines. We also report that new cancer therapeutic strategies are targeting KDM6B and EZH2, but the specificity of these treatments may be increased by learning more about the mechanisms of action of these enzymes and their specific partners or target genes in different cancer types.

## 1. Introduction

### 1.1. Epithelial to Mesenchymal Transition (EMT)

Epithelial to mesenchymal transition (EMT) is a phenomenon that leads epithelial cells to gradually acquire a mesenchymal phenotype. This process is associated with a loss of expression of specific epithelial proteins called “epithelial markers,” such as E-CADHERIN (coded by *CDH1* gene), CLAUDIN, or OCCLUDIN, and a gain of expression of other proteins called “mesenchymal markers,” such as VIMENTIN or N-CADHERIN. At least three distinct types of EMT have been reported so far, even if they present highly similar mechanisms: (i) type I is implicated in embryo formation and organ development; (ii) type II is involved in wound healing, regeneration of tissues, and in organ fibrosis; and (iii) type III is implicated in cancer development and aggressiveness. The latter also plays a role in tumor escape, impairing the formation of the immune synapse or leading to the overexpression of the immune checkpoint inhibitor PD-L1 (programmed death ligand 1) [1,2,3], and in metastasis formation [4,5]. Indeed, in type III EMT, tumor cells have been shown to present a loss of expression of adhesion proteins [6], to secrete various metalloproteases, such as MMP9 or ADAM19 [7], leading to the digestion of the extracellular matrix and then to the escape of the primary tumor and the migration through the blood or lymphatic circulation [4,5].

**Figure 1:**
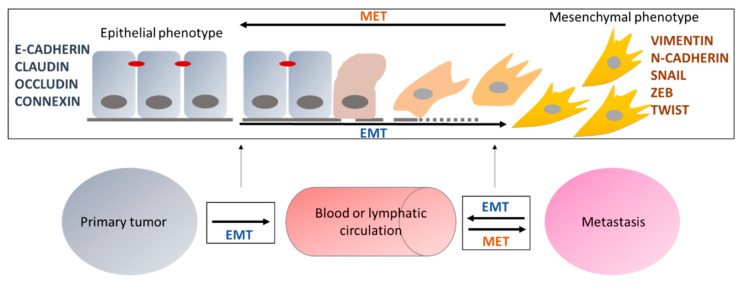
Roles of EMT in the formation of metastases. Epithelial cells, expressing epithelial markers, can undergo EMT and progressively acquire a mesenchymal phenotype characterized by the expression of mesenchymal markers. EMT is reversible and the opposite transition is called MET (mesenchymal to epithelial transition). EMT and MET are involved in the emergence of metastases by allowing cells to migrate to the blood or lymphatic circulation and then invade another organ to generate a secondary tumor called metastasis.

All the changes occurring during EMT at the morphological, functional, or molecular levels can be reversed to return to the original epithelial phenotype. This phenomenon is called mesenchymal to epithelial transition (MET) [8,9]. Through this process, circulating cancer cells can invade another organ, leading to the formation and growth of a secondary tumor called metastasis (Figure 1). However, the definition of EMT remains complex since there is not only one EMT but a multitude of intermediate states presenting different expressions of epithelial and mesenchymal markers. This plasticity has been described to be mainly linked to the epigenetic regulation of EMT, which is itself a highly plastic and reversible process, as described below.

Type III EMT can be induced by various intra- or extra-cellular factors or stress. It has been described that EMT-activating transcription factors (EMT-ATFs), such as SNAIL, TWIST, and ZEB (zinc finger E-box-binding homeobox) families, are main regulators of EMT [10]. These EMT-ATFs are activated via hypoxia, cytokines (TGFβ, TNFα), growth factors (EGF), or by cellular signaling pathways (WNT, HEDGEHOG, NF-κB, and NOTCH), and their activation leads to EMT induction [11].

### 1.2. Epigenetic Regulation of EMT

The word “epigenetics” was used for the first time in 1942 by Conrad Hal Waddington, who used the term of “epigenotype” to describe the different phenomena which are involved in the implementation of a phenotype from a genotype, and “epigenetics” for studies related to the understanding of this phenomenon [12]. This definition has been widely modified since the 1940s to lead to the one currently used now: “Epigenetics is the study of the regulation of gene expression, without DNA nucleotide sequence alteration, and linked to hereditary transmission.” This field includes the study of non-coding RNA (miRNA, lncRNA, etc.), which can bind DNA or mRNA and influence gene transcription, DNA methylation (methylation of cytosines in CpG regions leading to the repression of transcription) and histone post-translational modifications (Table 1). Plasticity of epigenetic regulation is conferred by the reversible character of the corresponding biochemical reactions. Indeed, DNA methylation, histone post-translational modifications, and non-coding RNA expression can be reversed according to the cell pathophysiological context. Moreover, epigenetic modifications have been widely linked to EMT regulation and suggested to confer the necessary plasticity to regulate cancer aggressiveness, invasion, and metastasis [13].

**Figure 2:**
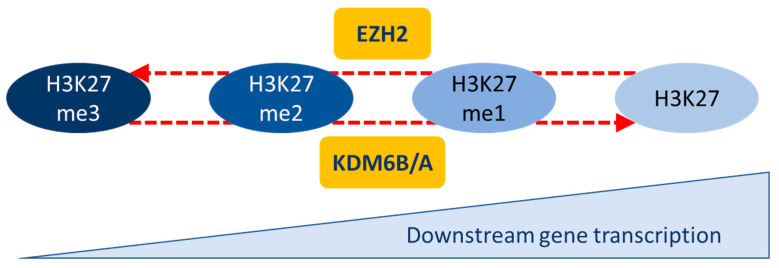
Regulation of H3K27 methylation status. H3K27 methylation status is regulated via three enzymes: EZH2 (methyltransferase) and KDM6B/A (demethylases). The H3K27 methylation level is inversely correlated to downstream gene transcription.

In this review, we will focus on the regulation of the methylation of lysine 27 of histone H3 (H3K27me3), a repressive mark, which has been described as a master regulator of EMT. It is noteworthy that the amino group of lysine 27 of histone H3 can also be acetylated and that the H3K27Ac mark is generally associated with increased gene transcription and therefore inversely correlated with H3K27me3 [14,15]. It has also been shown that the H3K27me3 mark is associated with temporarily repressed gene expression, which could easily be re-expressed later [16]. H3K27 can be mono-, di-, or tri-methylated (H3K27me, H3K27me2, and H3K27me3). H3K27me is associated with active transcription, whereas H3K27me2, and in particular H3K27me3, are associated with repressed genes (Figure 2). This methylation status is regulated via a histone methyltransferase, EZH2 (enhancer of zeste homolog 2), which mono-, di-, or tri-methylates H3K27; and two lysine demethylases, KDM6B (lysine demethylase 6B) and KDM6A (lysine demethylase 6A), which can mono-, di-, or tri-demethylate H3K27.

### 1.3. Current Described Roles of KDM6B and EZH2

**Figure 3:**
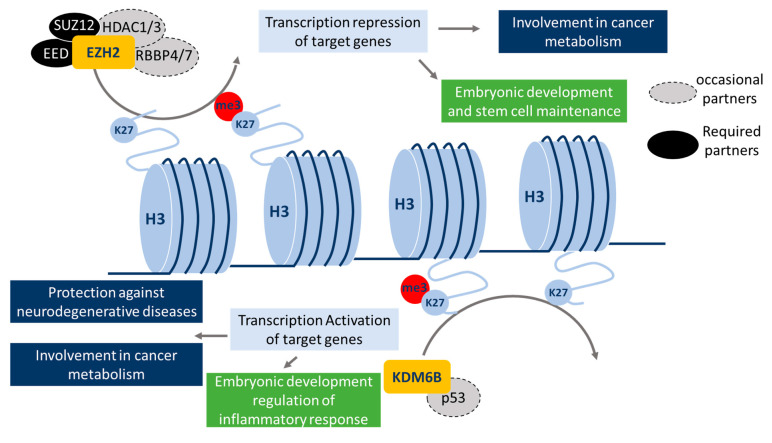
The current described roles of EZH2 and KDM6B. EZH2 methyltransferase activity, which catalyzes mono-, di-, or tri-methylation of H3K27, requires the presence of the PRC2-associated proteins, SUZ12 and EED. HDAC 1/3 and RBBP 4/7 can also be part of this complex but are not required for methyltransferase activity. EZH2 is important for normal embryonic development and stem cell maintenance. Moreover, it can also regulate cancer metabolism. KDM6B presents a lysine demethylase activity, which is a specific feature of H3K27. p53 has been reported to interact with KDM6B but is not required for its demethylase activity. KDM6B is required for embryonic development and the regulation of inflammatory responses, and is also important in cancer metabolism regulation and presents protective effects in some neurodegenerative diseases.

EZH2, also called KMT6A (lysine methyl-transferase 6A or ENX-1), is a subunit of the polycomb repressive complex 2 (PRC2), which is normally involved in embryonic development and in stem cell maintenance. PRC2’s main function is the di- or tri-methylation of H3K27, via its catalytic subunit EZH2. The other essential components of the PRC2 complex are SUZ12 (suppressor of zeste 12) and EED (embryonic ectoderm development). SUZ12 is directly involved in the tri-methylation of H3K27me3, more likely by inducing EZH2 binding to histone and modulating EZH2 conformation [17]. EED has a H3K27 binding domain that favors the recruitment of PRC2 on histone H3. Additional proteins have also been associated with the PRC2 complex: RBBP4 and RBBP7 (retinoblastoma binding protein 4 and 7), and HDAC1 and HDAC2 (histone deacetylase 1 and 2). However, these proteins are not required for the histone methyltransferase activity of PRC2 [18] but they may drive the recruitment of the PRC2 complex onto DNA or histone H3 (Figure 3). PRC2 can regulate different subsets of genes depending on the cell type. Indeed, it has been described that PRC2 may be recruited to chromatin on a response element (PRE = PRC2 response element) [19], but the cell type-dependent profiles of PRC2 recruitment remain unclear. However, the potential presence of specific DNA- (or histone)-binding proteins in the PRC2 complex (e.g., HDAC1 and 2 or RBBP4 and 7) may partially explain these cell type specificities (Figure 3).

KDM6B, also called JMJD3 (Jumonji domain containing protein 3), is a H3K27me3 lysine demethylase, which plays an important role in the regulation of embryogenic development via controlling *HOX* genes (a subset of homeotic genes). For example, KDM6B can directly demethylate H3K27me3 in *HOX* and *BMP* (bone morphogenetic protein) promoters leading to activated transcription, which is essential for bone differentiation [20]. KDM6B also plays a role in the regulation of the inflammatory response by controlling macrophage differentiation (transcription activation of *HOXA11* and *BMP2* genes) [21]. The proteins involved in the recruitment of KDM6B on chromatin are poorly documented. Nonetheless, it has been shown that p53 can bind to KDM6B and promotes the recruitment of the complex on its target genes to activate their transcription [22].

Furthermore, even if PRC2 can bind to its PRE, this is not sufficient to explain the target genes of EZH2, and KDM6B appears to not have any affinity to specific DNA regions. EZH2 and KDM6B may rather require protein partners to specifically bind chromatin (Figure 3).

The deregulation of KDM6B and EZH2 has been described to be involved in the development of several disorders. KDM6B seems to prevent some neurodegenerative diseases, such as Alzheimer’s disease [23], but the most documented disease linked to KDM6B and EZH2 dysregulation is cancer [23,24,25]. Indeed, both these enzymes have been involved in EMT and tumor aggressiveness in various cancer cell types, and this review will specifically focus on the roles of EZH2 and KDM6B on the epigenetic regulation of EMT. Despite their described antagonist roles, these two enzymes were almost never studied together, apart from the work from Daures et al. [26,27].

## 2. EZH2 Is Associated to EMT and Cancer Aggressiveness

### 2.1. EZH2 Is Differently Expressed between Tumor and Normal Tissues in Various Cancer Types

A great number of publications studied EZH2 expression in different tumors using various techniques. These data have been summarized in Table 2. In clear cell renal cell carcinomas (ccRCCs), a high expression of EZH2 has been correlated with bad prognosis, metastasis, and high grade tumors [28,29]. Similar observations were collected in esophagus squamous cell carcinoma, in which EZH2 expression levels are higher in tumors compared to adjacent tissues and this higher expression has been correlated to the apparition of distant metastasis, bad prognosis, depth of invasion, and tumor size [30]. Similar data were also obtained in bladder urothelial carcinoma; in cutaneous (human and murine) melanoma; in hormone refractory and metastatic prostate cancer; in gastric cancer; breast, lung, and ovarian carcinoma; as well as in pediatric brain tumors. Therefore, it is clear that a high expression of EZH2 was frequently correlated to high grade tumors, aggressiveness, and poor patient outcome [31,32,33,34,35,36,37,38,39,40].

Although EZH2 seems generally associated to aggressiveness, some inactivating mutations or deletions of the *EZH2* gene have also been reported in different cancers, suggesting an ambiguous role of EZH2 in these cancers. In lung adenocarcinomas, the *KRAS* mutation is a major oncogenic mutation frequently associated with the *EZH2* mutation. Inactivating *EZH2* mutations was found in 14% of all the studied tumors. Conditional *EZH2* knock-out (KO) mice were also used to demonstrate that *EZH2* inactivation increased lung adenocarcinoma aggressiveness in mice carrying the *KRAS* mutation [41]. Moreover, a study performed on 119 patients with myelodysplastic syndromes and myeloproliferative neoplasms showed that 8.4% of the patients presented an inactivating mutation in the *EZH2* gene and that 3.4% of patients presented a deletion of this gene. Moreover, the deletion of the *EZH2* gene in mice was sufficient to induce a myeloid dysplasia [42].

### 2.2. EZH2 Modulates EMT by Targeting a Specific Subset of Genes

Since EZH2 was described to be overexpressed in many aggressive tumors, this protein was proposed to act as a master regulator of the expression of EMT ATFs. However, it has been shown, in head and neck squamous cell carcinoma cell lines FaDu and SNU1041, that EZH2 did not regulate *SNAI1* or *SNAI2* gene expression. Moreover, a siRNA-driven knock-down of *EZH2* increased E-CADHERIN levels and decreased N-CADHERIN and VIMENTIN levels, leading to an inhibition of migration and invasion capacities without modulating EMT ATFs levels [43]. Surprisingly, in the ovarian cancer cells SKOV3, ChIP experiments showed that EZH2 could directly repress *ZEB2* expression when EMT was induced in these cells following TGF-β treatment [44] suggesting that EZH2 inhibited EMT in these cells. In the MHCC97H and MHCC97L cells lines (hepatocellular carcinomas), lncRNA *Carlo5* bound to EZH2 protein and recruited it onto the *miR-200b* promoter to repress its transcription. *miR-200b* was previously described to be linked to an epithelial phenotype by blocking EMT, provoked by the inhibition of ZEB1 and ZEB2 signaling (repressors of *CDH1* gene transcription). The repression of *miR-200b* expression by the complex lncRNA *Carlo5*-EZH2 therefore favored EMT [45]. In non-small cell lung cancer cells, *miR-21* overexpression also enhanced *EZH2* expression via an unknown mechanism and promoted cell invasion and migration [46].

Even if the implication of EZH2 in the regulation of EMT ATFs expression is not clearly defined, many interactions between these proteins have been reported. Indeed, overexpression of TWIST1 in bone marrow squamous cells led to the overexpression of *EZH2* via an unknown mechanism. This was also associated with the repression of cell proliferation mediated by the recruitment of EZH2 and addition of the H3K27me3 marks on the *P16/INK4/ARF* locus [47]. In oral tongue squamous cell carcinomas cells, Cal27, SNAI1, and SNAI2 induced the repression of *miR-101*, and consequently increased the levels of EZH2 in an unclear mechanism [48]. In the nasopharyngeal carcinoma cell line, HNE1 and SNAI1 can bind to the deacetylase enzymes HDAC1/2, which has also been described to interact with EZH2. This linear repressive complex is then able to trimethylate H3K27 and deacetylate histones, and thus repress the transcription of target genes, such as *CDH1* [49].

EZH2 did not only regulate EMT via its interaction with EMT ATFs, but also by regulating several target genes that were described as EMT regulators in many cell types (Table 3). Indeed, the down-regulation of *CDH1* can be directly controlled via EZH2. It has been shown in the colon cancer cell line HT-29-M6 that both SNAI1 and EZH2 were required to recruit PRC2 on the promoter of *CDH1*, and that EZH2 and SNAI1 stabilized each other on this promoter [50]. Cho and collaborators also reported that EZH2 directly repressed *CDH1* expression by the trimethylation of H3K27 on its promoter [51]. Similarly, it has been suggested, in the ovarian cancer cell line HO-8910, that EZH2 repressed *CDH1* gene expression. Indeed, a siRNA-driven knock-down of *EZH2* led to an increase in E-CADHERIN expression and to a decrease in invasion and migration capacities of these cells [36]. In the prostate cancer cell line PC3, the chemical compound triptolide (a promising anti-cancer drug whose mechanisms are not well described but are believed to involve the regulation of caspases, NF-κB pathway, heat-shock proteins, and DNA repair associated factors [52]) decreased mRNA levels of *EZH2*, and led to an increase in *CDH1* expression [53].

RKIP (Raf-1 kinase protein inhibitor) inhibited tumor metastasis in different types of cancers such as breast, prostate, gastric, and cervical cancers [54,55,56]. In breast (MCF-7, T47-D, and MDA-MB-231) and prostate (LNCaP, DU145, and PC3) cancer cell lines, EZH2 directly repressed *RKIP* transcription by inducing H3K27 trimethylation on its promoter, leading to increased metastasis [57]. Furthermore, in melanomas, EZH2 similarly repressed *AMD1* gene expression (a protein involved in polyamine synthesis), leading to increased levels of SNAI1, ZEB1, and TWIST1, and to increased invasion and metastasis, both in vitro and in vivo [40]. In small cell lung cancers, EZH2 overexpression is related to poor prognosis and associated to the H3K27me3-dependent repression of *JUB1* in these tumors. Moreover, overexpression of JUB1 in small cell lung cancer cells—DMS53, Lul30, and H209 cells—decreased cell proliferation showing that EZH2 promoted lung cancer cell proliferation via the inhibition of JUB1 expression in these models [58]. *P27^kip^*, a gene coding a tumor suppressor protein involved in cell cycle arrest via the inhibition of CDK proteins, presented an EZH2-dependent repression of transcription in MIA-PaCa2 and Panc04.03 cancer cells, leading to uncontrolled cell growth and aggressiveness [59].

EZH2 also promoted EMT and cancer aggressiveness via the repression of genes involved in different signaling pathways. Indeed, in liver cancers, EZH2 was recruited by the long non-coding RNA *lncAPC* to the *APC* promoter where it repressed transcription in a H3K27 methylation-dependent manner. APC normally participates to the cytosolic sequestration of β-CATENIN and to its degradation. EZH2 overexpression therefore led to the activation of the Wnt/β-CATENIN pathway that favored liver tumor initiating cells (TIC) self-renewal and cancer aggressiveness [60]. In the non-small cell lung adenocarcinoma cell lines, A549 and H358, chemical inhibition (using the specific GSK126 inhibitor) of EZH2 led to increased levels of *IGF1* mRNAs, associated to a global decrease in H3K27me3 levels [41]. Amongst the IGF1 downstream targets, AKT and ERK signaling pathways were previously described to be both associated to increased proliferation.

Data obtained in breast cancers also illustrated the dual role of EZH2 in cancer aggressiveness. In the MDA-MB-231 cell line, an ER (estrogen receptor)-negative cell line, EZH2 positively activated the transcription of NF-κB target genes in a methyltransferase-independent way, which contributed to aggressiveness of these breast cancer cells. On the contrary, in the ER-positive breast cancer cell line, MCF-7, EZH2 interacted with ER and activated the recruitment of the PRC2 complex on the NF-κB promoter where EZH2 tri-methylated H3K27 and repressed NF-κB transcription [61].

Altogether, these studies highlighted that EZH2 can be considered as an inducer of EMT or cancer aggressiveness in a large range of tumors, but surprisingly, in rare cases, EZH2 was also associated to anti-tumor properties. These studies highlighted the paradoxical role of EZH2 in the regulation of EMT.

### 2.3. EZH2 Modulates EMT and Cancer Aggressiveness via Activating Gene Transcription, Independently of Its Methyltransferase Catalytic Activity

As described above, EZH2 might present both methyltransferase and polycomb-independent activities (Table 3). In the absence of ER, in ER-negative breast cancers, EZH2 interacted with RelA and RelB, two NF-κB subunit proteins, but surprisingly, EZH2 mutants lacking the enzymatic domain were still able to interact with both these proteins showing that these interactions were independent of the catalytic subunit of EZH2. Furthermore, in cells overexpressing either an inactive or an active EZH2, without expressing wild type EZH2, the transcription of NF-κB target genes was up-regulated, showing that EZH2 activated transcription of these genes independently of its catalytic activity. Moreover, co-immunoprecipitation experiments of SUZ12 confirmed the interaction of SUZ12 with EZH2, but not with RelA or RelB, data which strongly suggested that the binding of EZH2 with the PRC2 polycomb complex or RelA/RelB was exclusive. These data therefore suggested that the Polycomb complex was not required for the transcriptional role of EZH2 [61].

Similarly, in the breast cancer cell lines, MCF-7 and T47D, co-IP and GST-pulldown experiments revealed that EZH2 was found to interact with ERα and β-CATENIN and to induce the transcription of their target genes. In these models, these interactions contributed to cell cycle progression and cancer aggressiveness [62]. These examples also strongly suggested that EZH2 was able to induce the transcription of target genes independently of its interaction with the PRC2 complex.

### 2.4. Regulation of EZH2 Expression during EMT

*EZH2* expression was described to be regulated at both transcriptional and post-translational levels. Two different transcription factors have been reported to activate *EZH2* transcription during EMT. First, in the breast epithelial cancerous and normal cells, PY2T and NMuMG, SOX4 was described as a direct activator of *EZH2* transcription following TGF-β treatment [63]. Second, in the breast cancer cell lines—MDA-MB-468, MDA-MB-231, and SKBr3—the MEK-ERK1/2 pathway activated *EZH2* transcription via the direct recruitment of P-ELK1 on the *EZH2* promoter. Indeed, this activation was not observed in the less aggressive breast cancer cell line HCC1500 (ER-positive cells) [64].

Many miRNAs were also described to target *EZH2* mRNA leading to decreased cellular levels of *EZH2* mRNA. Among these miRNAs, *miR-101* was the most frequently observed, since it has been described to target *EZH2* in gastric cancer cell lines (BGC-823, SGC-7901, AGS, and MKN-45), in head and neck squamous cell carcinomas (HNSCCs) and in the glioblastoma cell line U87. In all these cancer models, *miR-101* inhibited migration, invasion, or EMT [65,66,67]. Similarly, in the hepatocellular carcinoma cell line HEPG2, another *miRNA*, *miR-137,* targeted *EZH2* mRNA that led to decreased cell migration and invasion. Indeed, *miR-137* was described to be less expressed in HCCs than in normal tissues [68]. Moreover, *miR-138* also inhibited EMT by targeting *EZH2* mRNA in the HNSCCs 1386Ln and 686Tu cell lines [69]. *miR-124* targeted the 3’-UTR of *EZH2* mRNA leading to its degradation and overexpression of this miRNA suppressed cell motility, invasion and EMT in vitro, and intra-hepatic and pulmonary metastasis formation in vivo [70]. In the esophageal squamous cell carcinoma cell line, Ecal09, *miR-98*, and *miR-214* also targeted the 3’-UTR of *EZH2* mRNA and inhibited migration and invasion. These miRNAs were downregulated in esophageal squamous cell carcinomas and their expression was inversely correlated with lymph node metastasis [71]. Finally, various miRNA targeted *EZH2* mRNA and inhibit migration and invasion in various cancer cell lines. *MiR-214*, *miR-4465*, and *miR-137* targeted EZH2 respectively in hepatocellular carcinoma cell line SK-HEP-1, in non-small cell lung cancer cell lines A549 and H2170, and in melanoma cell lines Ma-Mel-79b and -86b [72,73,74]

Altogether, these data demonstrated that the *EZH2* gene and *EZH2* mRNA levels are tightly regulated by both transcription factors and miRNAs during EMT.

### 2.5. New Anti-Cancer Therapeutic Protocols Targeting EZH2 Activity

More than 10 clinical studies are evaluating the effects of EZH2 inhibitors (EZH2i) against multiple cancers (https://clinicaltrials.gov). For example, a phase I study using the EZH2 chemical inhibitor GSK2816126 in elapsed/refractory diffuse large B-Cell lymphoma (DLBCL), other non-Hodgkin lymphomas (NHL), transformed follicular lymphoma (tFL), solid tumors, and multiple myeloma (MM) showed an evident anti-tumoral effect of the molecule [75]. Many different EZH2i are tested such as tazemetostat (used in almost 15 studies), MK683, CPI-1205, and SHR2554. Although these compounds are still being evaluated, it appears that EZH2 inhibition is a very promising therapeutic approach in a wide range of cancer types (solid tumors or not) (Table 4).

## 3. KDM6B in EMT and Cancer Aggressiveness

### 3.1. KDM6B Is Differently Expressed between Tumor and Normal Tissues in Various Cancer Types

KDM6B has been described to be differentially expressed between cancer and normal adjacent tissues (Table 2). For example, in hepatocellular carcinomas, tissue-based RT-qPCR and Western-blotting analysis showed that *KDM6B* was overexpressed in tumor tissues compared to normal adjacent tissues. Moreover, *KDM6B* expression was correlated with distant metastasis and with an increased expression of *SNAI2* [76]. In clear cell renal cell carcinomas, KDM6B expression was also higher in tumor tissues compared to normal ones (tissue-based RT-qPCR, Western blotting, and IHC), and overexpression of KDM6B was also linked to increased tumor size, metastasis, and poor prognosis for patients [77]. Similar results were observed in multiple myelomas according to a public data set study [78] and in Hodgkin’s lymphomas (IHC analysis) [79]. An *Oncomine* data analysis also confirmed that KDM6B was overexpressed in invasive and metastatic breast tumors compared to less invasive tumors [80]. Similarly, RT-PCR data showed that *KDM6B* mRNA was overexpressed in malignant pleural mesothelioma (MPM) compared to benign pleura [81]. Moreover, KDM6B was also overexpressed in ovarian cancers at both protein and mRNA levels and its expression was associated with invasion, metastasis, and low overall survival [82]. All these studies therefore clearly indicated that KDM6B could be classified as an oncogene and was correlated to EMT and cancer aggressiveness. However, some contradictory or intriguing results have also been reported. For example, even if *KDM6B* was overexpressed in pancreatic ductile adenocarcinoma (PDAC) compared to normal tissue, its expression decreased with tumor grades [83]. Moreover, *KDM6B* was under-expressed in poor differentiated tumors (mesenchymal phenotype) in a cohort of 96 colon cancer patients [84]. In 20 colorectal cancer patients, *KDM6B* was also under-expressed compared to normal tissues, and a high KDM6B expression was correlated with better overall survival in a cohort of 151 colorectal cancer (CRC) patients [85]. Altogether, these data suggested that KDM6B could be correlated, or inversely correlated, with invasion and cancer aggressiveness regarding the cancer model. All these data are summarized in Table 5.

### 3.2. KDM6B Modulates EMT by Targeting a Specific Subset of Genes

KDM6B was described to modulate the expression of EMT ATFs during EMT in various cancer cell lines (Table 6). Indeed, the expression of SNAIL was directly activated by KDM6B. For example, in the mammary mouse cell line, NmuMG, H3K27 was demethylated by KDM6B on the *SNAI1* promoter when KDM6B was overexpressed, leading to the expression of mesenchymal-related genes [80]. In the hepatocellular carcinoma cell line, HepG2, KDM6B directly activated *SNAI2* transcription via a demethylation of H3K27 on its promoter and KDM6B overexpression promoted EMT, migration and invasion capacities and stem cell-like features (holoclone formation, clonogenic capacities, and sphere establishment) [76]. In the clear-cell renal cell carcinoma cell line, Caki-2, KDM6B also directly activated *SNAI2* transcription to increase mesenchymal marker expression, decreased epithelial protein expression, and increased invasion capacities [77]. In the renal cell carcinoma cell line, ACHN, the siRNA-driven knock-down of lncRNA *HOTAIR* led to decreased levels of both KDM6B and its target SNAIL1, and then decreased invasion and migration capacities of the cells [86], suggesting that KDM6B was also regulated at the mRNA level.

On the contrary, the knock-down of *KDM6B* or the expression of an inactive KDM6B mutant in SW-480 human colon cancer cells increased the expression of SNAIL1, ZEB1, and ZEB2 EMT ATFs, and led to consecutive decreases in E-CADHERIN, CLAUDIN1, and CLAUDIN7 expressions (epithelial markers) [84]. These data suggested that the KDM6B demethylase activity was frequently associated to EMT-related phenotypes, but some apparent contradictory roles of this enzyme could also be dependent on the model.

KDM6B has been described to modulate the expression of various genes involved in epithelial or mesenchymal cell state. Indeed, in the colorectal carcinoma cell line, HCT116, KDM6B directly activated the expression of the epithelial protein EPCAM (epithelial cell adhesion molecule) via its demethylase activity, leading to increased tumor growth, cell proliferation, and more surprisingly, to cell migration and invasion [87]. However, it should be noted that EpCAM and WNT/β-catenin expressions were inversely correlated with survival prognosis for patients with colon cancer [88]. In ovarian cancers, HER2 (human epidermal growth factor receptor 2, a receptor involved in proliferation, migration, and survival) and KDM6B expressions were correlated to poor prognosis and a low survival rate. KDM6B promoted EMT in the SKOV-3 ovarian cancer cell line and directly activated *HER2* gene transcription. Treatment of ovarian cancer cells with both Paclitaxel (which blocked the depolymerization of microtubule assembly in the mitotic spindle) and Carboplatin (a DNA alkylation chemical) decreased KDM6B content and was linked to an increase of the H3K27me3 mark on the *HER2* promoter leading to its decreased expression and decreased invasive and migratory cell capacities [89]. In the breast cancer cell lines, MDA-MB-231 and MCF7, the inhibition of the KDM6B demethylase activity, using the specific inhibitor GSKJ4, led to a repression of the expression of the multipotent *SOX2*, *NANOG*, and *OCT4* genes, a decrease of which was linked to an increase of the H3K27me3 mark on their promoters. These transcription factors have been described to be essential for the maintenance of the stem-like phenotype, suggesting that KDM6B regulated stemness properties of breast cancer cells [90]. On the contrary, KDM6B also promoted an epithelial-like phenotype, since, in the pancreatic ductal adenocarcinoma cell line BxPC3 and in xenograft models, KDM6B directly induced the demethylation of the H3K27me3 mark on the *CEBPA* promoter and induced the expression of this gene. Moreover, both knock-out of *KDM6B* or *CEBPA* led to the appearance of a mesenchymal phenotype and increased invasion capacities of these cells [83]. In the BJ foreskin cell line, KDM6B was recruited on a p53 binding element via its direct interaction with p53 where it demethylated the H3K27me3 mark. Indeed, KDM6B was proposed to participate to p53 target genes up-regulation and therefore, to cell cycle arrest or apoptosis and decreased cancer aggressiveness [22].

Some signaling pathways closely linked to EMT were also described to be regulated by KDM6B. In the melanoma A375-LM3 cell line, KDM6B demethylated H3K27 at the *NF-κB* and *BMP* promoters leading to the activation of NF-κB and BMP signaling target genes and thus contributed to the malignant cell properties. Moreover, the BMP signaling pathway activated KDM6B transcription via a positive feedback regulation loop [91]. In multiple myeloma, *KDM6B* was also induced via the NF-κB pathway and activated the MAPK pathway through its direct recruitment onto the *ELK1* and *FOS* promoters leading to induced cell growth, proliferation, and aggressiveness [78]. On the contrary, in human fibroblasts, KDM6B directly activated *P16/INK4A* gene transcription via demethylation of H3K27 on its promoter leading to a P16-dependent arrest of the cell cycle. [92,93]. Similar results were also described in the human lung fibroblast cells, TIG-3, after induction of *KDM6B* expression following the BRAF pathway activation. Indeed, KDM6B was able to block cell proliferation in these cells following its recruitment on the *P16/INK4A* promoter [94]. All these data are summarized in Table 5.

### 3.3. KDM6B Modulates Cancer Aggressiveness by Activating Gene Transcription, Independently of Its Demethylase Catalytic Activity

KDM6B can also modulate cancer aggressiveness in a demethylase-independent manner. In the immortalized human embryonic kidney cell line, HEK-293, KDM6B favored the expression of p53 target genes without being recruited on their promoters, but rather through increasing p53 recruitment on its target sequences in these promoters. Indeed, overexpression of KDM6B induced the nuclear localization of p53 in these cells and the recruitment of p53 on the *p21* promoter, but not of KDM6B at this site. These data were in favor of a tumor suppressor role of KDM6B via controlling the expression of p53 downstream genes [95]. This mechanism has also been observed for other targets, since KDM6B was involved in the stabilization of the transcription factor FOXO1 (Forkhead box protein O1, an inducer of pro apoptotic genes *BIM, TRAIL*, and *FasL*) in the nucleus of A549 and H460 non-small cell lung cancer cell lines. This interaction led to increased apoptosis and decreased migration and invasion [96] (Table 6).

### 3.4. New Anti-Cancer Therapeutic Protocols Targeting KDM6B Activity

Some pre-clinical studies already used the KDM6B inhibitors, GSKJ1/GSKJ4, to target cancers. GSKJ4 is a pro-drug for a cellular use of GSKJ1 when GSKJ1 is used in vitro This inhibitor has been identified by Kruidener and collaborators in 2012 [97]. In the acute lymphoblastic leukemia (ALL) cell line CUTLL1, GSKJ4 treatment induced cell cycle arrest and apoptosis and therefore is proposed as a promising therapeutic approach. However, this treatment did not affect myeloid leukemia cells, stromal cells, or hematopoietic progenitors [98]. GSKJ4 treatment also decreased tumor growth of xenografts of SF7761 and SF8628 pediatric brainstem glioma cells in mice. These cell lines present a heterozygous mutation of K27 of histone H3 (K27S) and half of histone H3 cannot be methylated at lysine 27 anymore. Nevertheless, mutated histone H3 sequestrated PRC2 and induced a dominant negative effect via reducing H3K27me3 on non-mutated histone H3. GSKJ4 treatment allowed the restoration of the mark H3K27me3 on histone H3, which was not mutated. GSKJ4 did not present the same effect on other pediatric brainstem cell lines, which did not contain the mutation [99].

## 4. Conclusions

This review highlights the paradoxical functions of EZH2 and KDM6B during EMT and cancer aggressiveness. Indeed, even if it has been shown that these proteins often present pro-EMT or pro-metastasis roles in a wide range of cancers, some examples revealed that they also presented anti-tumor functions in some cancers. Moreover, EZH2 and KDM6B were frequently both overexpressed and associated with EMT, invasion, migration, or metastasis in the same cancer types, such as ovarian cancers [36,82] or clear cell renal cell carcinomas [28,29,77]. These observations suggested that EZH2 and KDM6B expression might be regulated via shared similar mechanisms. However, to our knowledge, no studies targeted the common regulation of EZH2 and KDM6B during EMT and cancer. This review shows that the action of these two antagonistic enzymes during EMT is a complex phenomenon. The first paradoxical role of these enzymes is so that they are antagonistic, but both are involved in cancer aggressiveness and EMT sometimes in the same cell lines. The second paradoxical role is that these enzymes are also inactivated or under-expressed in some cancer types and still linked to epithelial phenotype in other cancer cell lines.

Therefore, all these studies suggested that EZH2 and KDM6B can be considered as master regulators of EMT through orchestrating the regulation of the H3K27me3 epigenetic mark. However, the mechanisms involved in this regulation remain unclear and seem often indirect and/or cell/cancer model-dependent. We think that the process called “type III EMT” gathers a lot of processes regulated by different pathways but lead to a common phenotype: the activation of EMT-ATFs expression and induction of a mesenchymal phenotype at both molecular and functional levels. We also know that cancer cells present a very variable genotype and that EZH2 and KDM6B could drive a specific cell phenotype depending on the cellular context. Moreover, interaction of EZH2 and KDM6B with different partners may also regulate their specific recruitment on promoters. Finally, H3K27me3 seems to be dynamically regulated during EMT and a slight change in the equilibrium between EZH2 and KDM6B expression, or activity, might rapidly modify the expression of the target genes. As described before, many studies have already focused on the regulation of EZH2 but the regulation of KDM6B remains largely unknown and may become a major issue to understand its function during EMT.

Therefore, the characterization of the role of EZH2 and KDM6B during EMT should provide new possibilities for designing anti-cancer therapeutic approaches. For example, in a recent study, Tazemetostat (an EZH2 chemical inhibitor which is used in many clinical trials) is more effective to target B-cell lymphoma cell lines models bearing EZH2 activating mutations than models bearing wild-type EZH2 since cell viability is less dependent on EZH2 in WT cells. Indeed, EZH2 inhibition led to the activation of B-cell pathway. A better understanding of the EZH2 target genes allowed the authors to associate Tazemetostat with B-cell pathway inhibitors to increase the specificity and the efficacy of this treatment in B-cell lymphoma with wild-type EZH2 [100].

## Figures and Tables

**Table 1:** Main histone post-translational modifications and their effects on gene expression.

Type of Histones Modification	Description	Effect on the Gene Expression
Acetylation	Acetylation of the lysine amino groups of histones	Decrease of histone/DNA interaction, chromatin is structurally loose, less compact, and transcription is activated
Methylation	Methylation of arginine or lysine in histones	Arginine: transcription activation; lysine: activation or inhibition of transcription depending on the lysine targeted and the number of methyl groups added (mono-, di-, or tri-methylation)
Ubiquitination	Mono-ubiquitylation of lysine of histones H2A and H2B	Regulation of transcription initiation and elongation
Phosphorylation	Phosphorylation of serine, threonine or tyrosine of histones	DNA damage response (phosphorylation of H2AX), modulation of DNA compaction and interaction with other histone post translational modifications
SUMOylation	SUMO protein conjugation on lysine of histones	Competition with other lysine modifications. Decrease and stop of transcription
ADP ribosylation	Ribosyl-ADP addition on lysine of histones	DNA damage and transcription activation
Arginine Citrullination	Transformation of an arginine into a citrulline residue	Decrease of DNA compaction
Proline isomerization	Isomerization of a proline in cis conformation into a trans conformation	Modulation of histone methylation and transcription

**Table 2:** EZH2 expression in various cancer types and correlation to clinical parameters. EZH2 is often overexpressed in cancers (green), but in a few cases such as lung adenocarcinoma or myelodysplastic syndromes, some inactivating mutations (red) have been found in the *EZH2* gene. IHC: immunohistochemistry; IF: immunofluorescence.

Cancer Type	Over/Under Expressed	Technique	Ref.
Clear cell renal cell carcinomas (ccRCC)	Overexpressed in metastasis	IHC	[29]
Clear cell renal cell carcinomas (ccRCC)	Overexpressed in aggressive and invasive carcinomas	IHC	[28]
Esophagus squamous cell carcinomas	Overexpressed	IHC	[30]
Bladder urothelial carcinomas	Overexpressed in invasive and aggressive carcinomas	RT-qPCR on cell lines and tissues	[39]
Urothelial carcinomas	Overexpressed	Tissue microassays/IHC	[38]
Cutaneous melanomas and cancers of the endometrium, prostate and breast	Overexpressed	IHC, Tissue microarray	[31]
Human and murine melanomas	Overexpressed	IF	[40]
Hormone refractory, metastatic prostate cancers	Overexpressed	Gene expression profiling	[37]
Gastric cancers	Overexpressed and correlated with aggressiveness and invasion	IHC	[35]
Breast carcinomas	Overexpressed in invasive tumors	Tissue microarray	[34]
Ovarian carcinomas	Overexpressed	IHC	[36]
Lung Adenocarcinoma	Overexpressed in poor prognosis tumors	IHC	[32]
Adult and pediatric brain tumors	Overexpressed	IHC	[33]
Lung adenocarcinomas	Inactivating mutations in 14% of the cohort samples	Sequencing	[38]
Myelodysplastic syndromes/Myeloproliferative neoplasms	Inactivating mutations	Extensive mutation analyses	[42]

**Table 3:** EZH2 target genes in various cancer cell lines. EZH2 directly downregulated (red) many genes in a wide range of cancer cell lines, but in some cases, it also directly, or indirectly, activated (green) transcription of some genes such as NF-κB target genes, *C-MYC* or *CYCLIN D1*.

Tissue of Origin of the Cell Line	Name of the Cell Line	Genes Regulated	Direct/Indirect	Ref.
Head and neck squamous cell carcinoma	FaDu and SNU1041	Down-regulation of *N-CADHERIN* and *VIMENTIN*	Unknown	[43]
		Up regulation of *E-CADHERIN*		
Ovarian cancer	SKOV3 and Kuramochi	Down-regulation of *ZEB2*, *VIMENTIN*, *N-CADHERIN*	Direct	[44]
Colon cancer	HT-29 M6	Down-regulation of *CDH1*	Direct	[50]
Non-small cell lung cancer	A549	Down-regulation of *CDH1*	Direct	[51]
Ovarian cancer	HO-8910	Down-regulation of *CDH1*, Up-regulation of *TGF-β1*	Unknown	[36]
Prostate cancer	PC3	Down-regulation of *ADRB2*, *DAB2IP*, *CDH1* and *CDKN2A*	Unknown	[53]
Prostate and breast cancer	LnCAP, MCF7, DU-145 and LSHAR	Down-regulation of *RKIP*	Direct	[57]
Human and murine melanomas	XB2, Melan-a, HEK293T, B16F1 and B16F10	Down-regulation of AMD1, DCK and WDR19	Direct	[40]
Small cell lung cancer	DMS53, Lul30, H209	Down-regulation of *JUB*	Direct	[58]
Pancreatic cancer	MIA-PaCa2 and Panc04.03	Down-regulation of *p27^kip^*	Direct	[59]
Liver hepatocellular carcinoma	huh-7 and Hep-3B	Down-regulation of *APC*	Direct	[60]
Non-small cell lung carcinoma	H358 and A549	Down-regulation of *IGF1*	Unknown	[41]
		Down-regulation of *AKT* and ERK (via IGF1)	Indirect	
ER-negative breast cancer	MDA-MB-231	Down-regulation of NF-κB target genes	Unknown	[61]
ER-positive luminal like breast cancer	MCF7	Up-regulation of NF-κB target genes by binding of RelA and RelB	Direct	[61]
Breast cancer	MCF-7	Up-regulation of *C-MYC* and *CYCLIN D1*	Direct	[62]

**Table 4:** Summary of the clinical trials targeting EZH2 in cancers. Different molecules targeting EZH2 are used in clinical trials against a large range of solid and liquid tumors.

Title	Phase	Inhibitor	Cancer
A phase 1 study of SHR2554 in subjects with relapsed or refractory mature lymphoid neoplasms	Phase 1	SHR2554	Relapsed or refractory mature lymphoid neoplasms
A phase 1 study of the EZH2 inhibitor tazemetostat in pediatric subjects with relapsed or refractory INI1-negative tumors or synovial sarcoma	Phase 1	Tazemetostat	Rhabdoid tumors, INI1-negative tumors, Synovial sarcoma and malignant rhabdoid tumor of ovary
A phase II, multicenter study of the EZH2 inhibitor tazemetostat in adult subjects with INI-1-negative tumors or relapsed/refractory synovial sarcoma	Phase 2	Tazemetostat	Malignant rhabdoid tumors, rhabdoid tumors of the kidney, atypical teratoid rhabdoid tumors and eight more.
ProSTAR: A study evaluating CPI-1205 in patients with metastatic castration resistant prostate cancer	Phase 1/2	CPI-1205	Metastatic castration resistant prostate cancer (mCRPC)
ORIOn-E: A study evaluating CPI-1205 in patients with advanced solid tumors	Phase 1/2	CPI-1205	Advanced solid tumors
Study of the EZH2 Inhibitor Tazemetostat in malignant mesothelioma	Phase 2	Tazemetostat	Mesothelioma, BAP1 loss of function
Open-label, multicenter, phase 1/2 study of tazemetostat (EZH2 histone methyl transferase (HMT) inhibitor as a single agent in subjects with advanced solid tumors or with B-cell lymphomas and Tazemetostat in combination with Prednisolone in subjects with DLBCL	Phase 1/2	Tazemetostat	B-cell lymphomas, advanced solid tumors, diffuse large B-cell lymphoma (and 3 more)
A study evaluating CPI-1205 in patients with B-cell lymphomas	Phase 1	CPI-1205	B-cell lymphoma
Tazemetostat in treating patients with relapsed or refractory advanced solid tumors, non-Hodgkin lymphoma, or histiocytic disorders with EZH2, SMARCB1, or SMARCA4 gene mutations (a pediatric match treatment trial)	Phase 2	Tazemetostat	Advanced malignant solid neoplasm, Ann Arbor stage II childhood Hodgkin lymphoma (and 47more)
A study to investigate the safety, pharmacokinetics, pharmacodynamics and clinical activity of GSK2816126 in subjects with relapsed/refractory diffuse large B-cell lymphoma, transformed follicular lymphoma, other non-Hodgkin’s lymphomas, solid tumors, and multiple myeloma	Phase 1	GSK2816126	Cancer
Safety and efficacy of MAK683 in adult patients with advanced malignancies	Phase 1/2	MAK683	Diffuse large B-cell lymphoma

**Table 5:** KDM6B expression in different cancer types. KDM6B is often overexpressed (green) in cancers; however, it was described to be under-expressed (red) in invasive pancreatic ductal adenocarcinomas and poor differentiated colon cancers.

Cancer Type	Over/Under Expressed	Technique	Ref.
**Hepatocellular carcinoma**	Overexpressed	Tissue RT-qPCR and Western blotting	[76]
**Clear cell renal cell Carcinoma**	Overexpressed	Tissue RT-qPCR, Western blotting, and IHC	[77]
**Multiple Myeloma**	Overexpressed	Public data set study	[78]
**Hodgkin‘s lymphoma**	Overexpressed	IHC	[79]
**Breast cancer**	Overexpressed	Oncomine data analysis	[80]
**Malignant pleural mesothelioma**	Overexpressed	Tissue RT-qPCR	[81]
**Ovarian cancer**	Overexpressed	IHC and tissue RT-qPCR	[82]
**Pancreatic ductal adenocarcinoma**	Under-expressed in invasive tumors compared to low grade cancers	Tissue microarray	[83]
**Colon cancer**	Under-expressed in poor differentiated tumors	Tissue RT-qPCR	[84]
**Colorectal cancer**	Under-expressed	IHC	[85]

**Table 6:** KDM6B target genes in various cancer cell lines. KDM6B directly activated (green) transcription of EMT-related genes in a wide range of cancer cell lines. It also down-regulated (red) the expression of EMT-ATFs, probably via an indirect way.

Tissue of Origin	Cell Line	Genes Regulated	Direct/Indirect	Ref.
Mouse breast	NmuMG	Up-regulation of *SNAI1*	Direct	[80]
Hepatocarcinoma	HepG2	Up-regulation of *SNAI2*	Direct	[76]
Clear cell renal cell carcinoma	Caki-2	Up-regulation of *SNAI2*	Direct	[77]
Renal cell carcinoma	ACHN	Up-regulation of *SNAI1*	Unknown	[86]
Colon carcinoma	SW-480	Down-regulation of *SNAI1*, *ZEB1*, and *ZEB2*	Unknown	[84]
Colorectal carcinoma	HCT116	Up-regulation of *EpCAM*	Direct	[87]
Ovarian cancer	SKOV-3	Up-regulation of *HER2*	Direct	[89]
Breast cancer	MCF-7 and MDA-MB-231	Up-regulation of *NANOG*, *SOX2*, *OCT3*	Unknown	[90]
Pancreas adenocarcinoma	BxPC3	Up-regulation of *CEBPA*	Direct	[83]
Skin, foreskin	BJ	Up-regulation of p53 target genes	Direct (via p53 binding)	[22]
Melanoma	A375-LM3	Up-regulation of *BMP2*; *BMP4*, *CCL2*, *STC1 TFPI2*, *PTGS2* and *TGM2*	Direct	[91]
Multiple myeloma	MM-1S	Up-regulation of *ELK1* and *FOS*	Direct	[78]
Lung	IMR-90	Up-regulation of *INK4a*	Direct	[92]
Lung fibroblast	TIG-3	Up-regulation of *INK4A*	Direct	[94]
Immortalized Human embryonic kidney cells	HEK-293	Stabilization of nuclear p53	Direct	[95]
Non-small cell lung cancer	H460 and A549	Stabilization of FOXO1 in nucleus	Direct	[96]

## Abbreviations

ADP	Adenosine Diphosphate
AKT	Protein Kinase B
ALL	Akute Lymphoblastic Leukemia
AMD1	Adenosyl Methionine Decarboxylase 1
APC	Adenomatous Polyposis Coli
ARF	Alternative Reading Frame
BIM	BCL2 Like 11
BMP	Bone Morphogenetic Protein
CDH1	Cadherin 1
CDK	Cyclin Dependant Kinase
CEBPA	CCAAT/Enhancer Binding Protein α
ChIP	Chromatin ImmunoPrecipitation
DLBCL	Diffuse Large B Cell Lymphoma
DNA	Desoxyribonucleic Acid
EED	Embryonic Ectoderm Development
EGF	Epidermal Growth Factor
EMT	Epithelial to Mesenchymal Transition
EMT-ATF	Epithelial to Mesenchymal Transition Activating Transcription Factors
EPCAM	Epithelial Cell Adhesion Molecule
ER	Estrogen Receptor
ERK	Extracellular Signal-Regulated Kinase
EZH2	Enhancer of Zeste Homolog 2
EZH2i	Enhancer of Zeste Homolog 2 Inhibitor
FOS	FBJ Murine Osteosarcoma Viral Oncogene Homolog
FOXO1	Forkhead Box Protein O1
H2A	Histone 2A
H2B	Histone 2B
H3	Histone 3
H3K27Ac	Histone 3, Lysine 27 Acetylated
H3K27me2	Histone 3, Lysine 27 Dimethylated
H3K27me3	Histone 3, Lysine 27 Trimethylated
H3K27me3	Histone 3, Lysine 27 Methylated
HCC	Hepatocellular Carcinoma
HDAC1	Histone Deacetylase 1
HDAC2	Histone Deacetylase 2
HER2	Human Epidermal Growth Factor Receptor
HOTAIR	HOX Transcript Antisense RNA
HOX	Homeobox
IF	ImmunoFluorescence
IGF1	Insulin-Like Growth Factor 1
IHC	Immunohistochemistry
INK4	Inhibitor of CDK4
JMJD3	Jumonji Domain-Containing Protein 3
JUB1	JUNGBRUNNEN 1
KDM6A	Lysine Demethylase 6A
KDM6B	Lysine Demethylase 6B
KMT6A	Lysine Methyltransferase 6A
KO	Knock-Out
KRAS	V-Ki-Ras2 Kirsten Rat Sarcoma Viral Oncogene Homolog
lncRNA	Long Non-Coding RiboNucleic Acid
MAPK	Mitogen-Activated Protein Kinase
MEK	Mitogen-Activated Protein Kinase
MET	Mesenchymal to Epithelial Transition
miRNA	Micro Ribonucleic Acid
MM	Multiple Myeloma
MPM	Multiple Pleural Mesothelioma
mRNA	Messenger RiboNucleic Acid
N-CAD	N-Cadherin
NF-κB	Nuclear Factor-κB
NHL	Non-Hodgkin Lymphoma
OCT4	Octamer-Binding Transcription Factor 4
PDAC	Pancreatic Ductile Adenocarcinoma
PD-L1	Programmed Death Ligand 1
PRC2	Polycomb Repressive Complex 2
PRE	Polycomb Repressive Complex 2 Response Element
RBBP4	RetinoBlastoma Binding Protein 4
RBBP7	RetinoBlastoma Binding Protein 7
RelA	V-Rel Avian Reticuloendotheliosis Viral Oncogene Homolog A
RelB	V-Rel Avian Reticuloendotheliosis Viral Oncogene Homolog B
RKIP	Raf-1 Kinas Inhibitor Protein
RNA	Ribonucleic Acid
RT-PCR	Reverse Transcription Polymerase Chain Reaction
RT-qPCR	Reverse Transcription Quantitative Polymerase Chain Reaction
siRNA	Small Interfering RiboNucleic Acid
SOX4	SRY-Related HMG Box
SUZ-12	Suppressor of Zeste 12
TF	Transcription Factor
tFL	Transformed Follicular Lymphoma
TGF-β	Transforming Growth Factor β
TIC	Tumor Initiating Cells
TNF-α	Tumor Necrosis Factor α
TRAIL	Tumor Necrosis Factor Related Apoptosis-Inducing Ligand
UTR	Untranslated Region
VIM	Vimentin
ZEB	Zinc Finger E-box Binding Homeobox
